# Upregulation of lnc-ZNF281 Inhibits the Progression of Glioma via the AKT/GSK-3*β*/*β*-Catenin Signaling Pathway

**DOI:** 10.1155/2021/5573071

**Published:** 2021-05-11

**Authors:** Yu-Qin Deng, Gang-Yong Kong, Song Li, Fen Li, Si-Lu Wen

**Affiliations:** Department of Otolaryngology-Head and Neck Surgery, Renmin Hospital of Wuhan University, 238 Jie-Fang Road, Wuhan, Hubei 430060, China

## Abstract

The purpose of this study is to elucidate the roles and potential underlying mechanisms of long noncoding RNA lnc-ZNF281 in glioma. We performed qRT-PCR to detect the expression levels of lnc-ZNF281 in glioma tissues. The effects of lnc-ZNF281 on the proliferative and migrative abilities of T98G and HS683 glioma cells were examined by cell proliferation assay, colony formation assay, wound-healing assay, and transwell assay. Also, the effects of lnc-ZNF281 on AKT/GSK-3*β*/*β*-catenin pathway were analyzed. The results showed that the expression of lnc-ZNF281 in glioma tissues was decreased compared with normal tissues. lnc-ZNF281 overexpression inhibited the proliferative and migrative abilities of glioma cells, while lnc-ZNF281 knockdown obtained the opposite findings. Besides, overexpression of lnc-ZNF281 in glioma cells inactivated the AKT/GSK-3*β*/*β*-catenin signaling pathway. Furthermore, *β*-catenin activation reversed the suppressive effects of lnc-ZNF281 on glioma cells. Taken together, lnc-ZNF281 inhibits glioma cell proliferation and migration via AKT/GSK-3*β*/*β*-catenin pathway and may serve as a potential target for glioma treatment.

## 1. Introduction

Glioma is one of the most prevalent and fatal brain tumors with low 5-year survival, approximately 5% [1]. Maximal resection followed by chemoradiotherapy is the currently standard treatment for glioma [2]. Recently, immunotherapy to PD-1 and CTLA-4 also exhibits promising results in glioma [3, 4]. The advances in glioma treatment really increase the survival of glioma patients, but with a high rate of recurrence and poor prognosis [2, 5]. However, the underlying mechanisms of highly invasive growth of glioma are unclear, and the approaches to overcome this obstacle are absent. Therefore, it is imperative to explore the underlying molecular mechanisms of glioma occurrence and progression to find more effective targets for glioma treatment.

Long noncoding RNAs (lncRNAs) are one type of RNA without protein-coding ability and have a length exceeding 200 nucleotides [6]. A growing number of studies have shown that lncRNAs play critical roles in many biological processes and signaling pathways just like protein-coding genes [7]. For example, lncRNA CRNDE activated Wnt/*β*-catenin signaling pathway and then promoted the proliferation and chemoresistance in colorectal cancer cells [8]. By interacting with the MYC gene, lncRNA EPIC1 promoted cell-cycle progression in gastric cancer cells [9]. Meanwhile, substantial dysregulated lncRNAs were identified to exert oncogenic or antitumor effects on glioma [10]. lncRNA H19 could bind to the 3′-UTR of miR-138 and then release the inhibitory effects of miR-128 on HIF-1*α* expression and VEGF. After that, increased HIF-1*α* and VEGF promoted glioma cell proliferation, migration, and angiogenesis [11]. lncRNA-ATB enhanced TGF-*β*-mediated invasion in glioma cells by modulating the activity of p38/MAPK and NF-*κ*B signaling pathways [12].

Among the known lncRNAs, lncRNA zinc finger protein 281 (lnc-ZNF281) is characterized as a tumor suppressor in osteosarcoma by downregulating the expression of miR-144 [13]. Besides, lnc-ZNF281 suppressed glioma stem-like U251s cells growth and invasion by regulating the NF-*κ*B signaling pathway [14]. However, upregulated lnc-ZNF281 was found in hepatocellular carcinoma tissues, and the interaction between lnc-ZNF281 and miR-539 played an oncogenic role in hepatocellular carcinoma progression [15]. lnc-ZNF281 also promoted gastric cancer initiation and progression by targeting miR-124 [16]. According to previous findings, the specific correlation between lnc-ZNF281 and glioma cells needs further exploration. Thus, we performed this research to elucidate the effects and mechanisms of lnc-ZNF281 on glioma.

## 2. Materials and Methods

### 2.1. Clinical Samples

Thirty glioma tissues and 22 normal brain tissues were collected from the Department of Neurosurgery of the Second People's Hospital of Huai'an City between January 2013 and November 2015. The diagnosis of glioma was confirmed by two or more experienced pathologists. After surgical removal, all samples were placed into liquid nitrogen for further experiments. Both the enrolled patients and healthy participants signed the written informed consent. The Research Ethics Committee of Renmin Hospital of Wuhan University approved this study.

### 2.2. Cell Culture

HS683 and T98G glioma cells were purchased from the American Type Culture Collection. All cells were cultured in DMEM (HyClone, Victoria, Australia), which contains 10% fetal bovine serum (FBS) and 1% antibiotics (penicillin–streptomycin, Gibco, California, USA). A humid environment with 5% CO_2_ and a temperature of 37°C was used for cell culture.

### 2.3. Plasmid Construction and Transfection

lnc-ZNF281 lentiviral overexpression vectors LV-lnc-ZNF281 and the negative control (NC) LV-NC were purchased from GenePharma Company (Shanghai, China). The small interfering RNA (siRNA) used to knock down lnc-ZNF281 (si-lnc-ZNF281) was also purchased from GenePharma. Cells were grown in 6-well plates and then transfected by lentiviral vectors according to the manufacturer's protocols. Transfected cells were treated by puromycin to establish stably transfected cell lines, and qRT-PCR was used to detect transfection efficiency. The transfection of si-lnc-ZNF281 was performed using the reagent kit (GenePharma) based in its instruction. Similarly, the transfection efficiency was detected at 48 hours after transfection.

### 2.4. qRT-PCR

Total RNAs were extracted from clinical samples and glioma cell lines by TRIzol Reagent (Invitrogen, CA, USA). The complementary DNA (cDNA) was synthesized from total RNAs using a Reverse Transcription Kit (Takara, Dalian, China). Then, quantitative real-time PCR (qRT-PCR) detection was performed using the SYBR Green real-time PCR Kit (Takara, Dalian, China). The expression of GAPDH was used as the internal reference. The sequences of specific primers were as follows: ZNF281 forward, 5′-GGACACATAGTGGAGAAAAG-3′ and reverse, 5′-GAGACAACACAGCCAGATTA-3′; *β*-catenin forward, 5′-AAAGCGGCTGTTAGTCACTGG-3′ and reverse, 5′-CGAGTCATTGCATACTGTCCAT-3′; GAPDH forward, 5′-GACTCATGACCACAGTCCATGC-3′ and reverse, 5′-AGAGGCAGGGATGATGTTCTG-3′. Every experiment was performed three times, and the 2^-∆∆Ct^ method was used to analyze all data.

### 2.5. CCK-8 Assay

The transfected cells were seeded in a 96-well microplate at a density of 3000 cells/well and then cultured overnight. After 1, 2, 3, or 4 days of incubation, 10 *μ*L Cell Counting Kit-8 (CCK-8, Beyotime Biotechnology, Shanghai, China) reagent was added to every well and cells were further cultured for another 2 hours at 37°C. In the SKL2001 (Selleck, Shanghai, china) activated group, this agonist was added into the medium to activate target cells. The optical density of each well at 450 nm was detected using a microplate reader (Thermo Fisher Scientific, MA, USA).

### 2.6. Colon Formation Assay

The transfected cells were seeded in a 6-well plate at a density of 1000 cells/well and cultured for 2 weeks at 37°C. Then, cell colonies were fixed with 4% paraformaldehyde for 20 minutes and stained with 0.1% Crystal Violet (Solarbio, Beijing, China) for 30 minutes. Finally, cell colonies with at least 50 cells were counted and photographed under an ordinary optical microscope (Olympus, DX51).

### 2.7. Transwell Assays

The transfected HS683 and T98G cells were cultured into the upper chamber in transwell inserts (Corning, NY, USA) at a density of 8 × 10^4^ cells with 200 *μ*l serum-free medium. The lower chambers were fixed with 500 *μ*l medium containing 10% FBS (HyClone, Victoria, Australia). After being incubated for 48 hours at 37°C, cells on the lower surface of the chamber were fixed with 4% paraformaldehyde and stained with 0.1% Crystal Violet, and cells on the upper surface were brushed. The chambers were placed under a light microscope and photographed to count cell numbers. In the SKL2001 (Selleck, Shanghai, China) activated group, this agonist was added into the serum-free medium to activate target cells.

### 2.8. Wound-Healing Assays

The transfected HS683 and T98G cells were passaged in a 6-well plate and grew to 90% of the whole well. Then, a 200 *μ*l pipette tip was used to create the straight wounds. Cells were washed using PBS for 3 times to eliminate cell fragments and unattached cells, and then, cells were cultured in a serum-free medium at 37°C. At 0 and 24 hours after the wounding, photographs were taken under a light microscope.

### 2.9. Western Blot

According to the protocol, total protein was extracted from transfected cells using RIPA buffer (GenStar, Beijing, China) and a bicinchoninic acid assay kit (GenStar, Beijing, China) was used to measure the concentration of protein. Equal amounts of protein samples were separated by 12% SDS-PAGE and then transferred onto a polyvinylidene fluoride membrane (Millipore, MA, USA). The membranes with primary antibodies specific for AKT (CST, 4961, Boston, USA), p-AKT (CST, 4070), GSK-3*β* (CST, 12456), p-GSK-3*β* (CST, 5558), *β*-catenin (CST, 8480), and GAPDH (Cell Signaling Technology, Danvers, MA, USA) were incubated overnight at 4°C. The membranes were incubated with corresponding horseradish peroxidase-conjugated secondary antibodies (Santa Cruz, CA, USA) at room temperature on the next day. The protein bands were then detected using an enhanced chemiluminescence detection kit (KeyGen, Nanjing, China).

### 2.10. Statistical Analysis

All experiments were performed 3 or more times. All data are expressed in the form of mean ± SD and analyzed using SPSS 19.0 (Chicago, IL, USA). Differences between groups were analyzed using unpaired Student's *t*-test or one-way ANOVA. It was considered a significant difference when *p* < 0.05.

## 3. Results and Discussion

### 3.1. Expression Level of lnc-ZNF281 Was Decreased in Glioma Tissues

**Figure 1:**
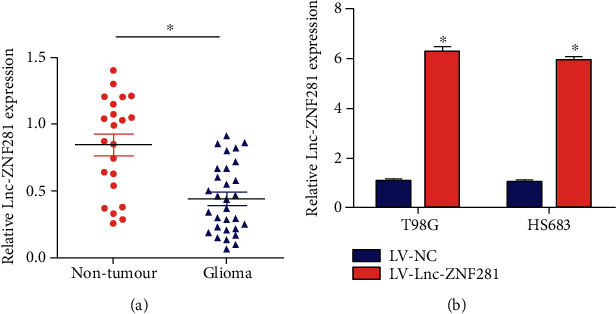
Expression levels of lnc-ZNF281 were reduced in glioma tissues. (a) qRT-PCR results indicated lnc-ZNF281 had a low expression level in glioma tissues compared to normal brain tissues. (b) qRT-PCR results indicated that the expression level of lnc-ZNF281 in glioma cells increased significantly after lentiviral transfection. ^∗^*p* < 0.05.

Firstly, we measured the expression level of lnc-ZNF281 in 30 glioma tissues and 22 normal brain tissues using qRT-PCR. The results showed that lnc-ZNF281 was lowly expressed in glioma tissues compared with normal brain tissues (Figure 1(a)). For subsequent experiments, we transfected T98G and HS683 glioma cells with lnc-ZNF281 lentiviral overexpression vectors LV-lnc-ZNF281 and the negative control vectors LV-NC. Then, we measured the expression level of lnc-ZNF281 in T98G and HS683 cells using qRT-PCR. The results indicated that the expression level of lnc-ZNF281 in glioma cells increased significantly (Figure 1(b)).

### 3.2. Overexpression of lnc-ZNF281 Suppressed the Proliferation and Migration of Glioma Cells

**Figure 2:**
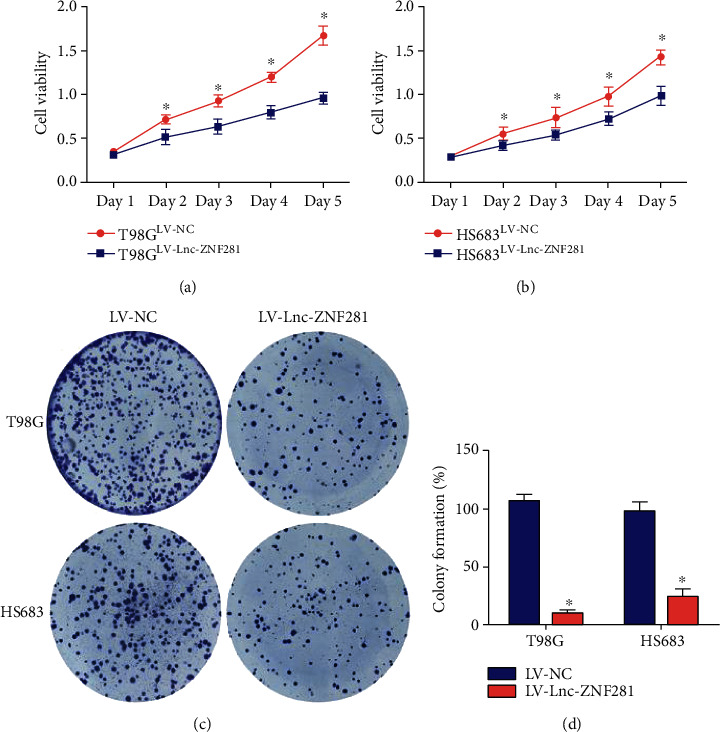
Overexpression of lnc-ZNF281 inhibited glioma cells proliferation. (a, b) The CCK-8 assay results indicated that overexpression of lnc-ZNF281 prevents the growth rate of glioma cells. (c, d) Cell colony formation assay results indicated that overexpression of lnc-ZNF281 inhibited the proliferation of glioma cells. ^∗^*p* < 0.05.

**Figure 3:**
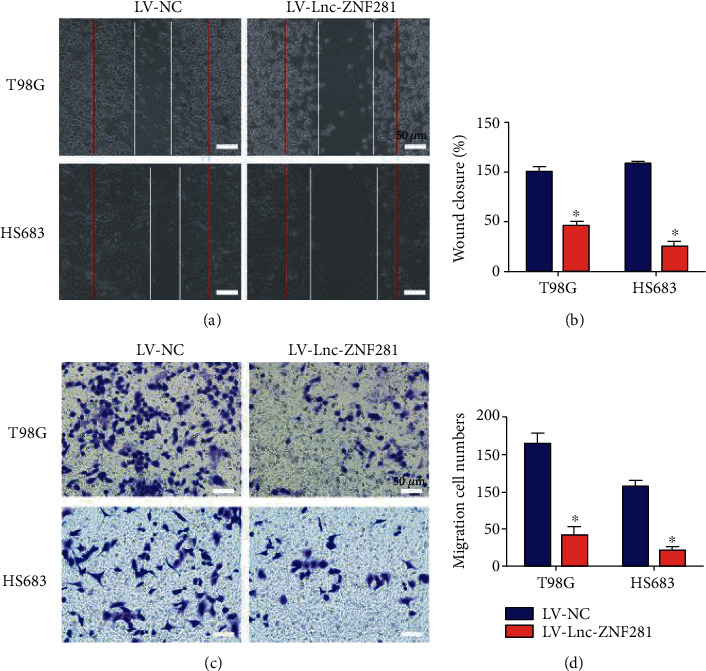
Overexpression of lnc-ZNF281 inhibited glioma cell migration. (a, b) The wound-healing assay results revealed that overexpression of lnc-ZNF281 inhibited cell migration in glioma. Images were acquired at 0 h (red line) and 24 h (white line). (c, d) The transwell assay results indicated that overexpression of lnc-ZNF281 inhibited cell migration in glioma. Scale bars = 50 *μ*m. ^∗^*p* < 0.05.

We performed CCK-8 cell proliferation assay and colony formation assay for assessing the effect of lnc-ZNF281 on the proliferation of glioma cells. The CCK-8 assay results showed that overexpression of lnc-ZNF281 decreased the growth rate of glioma cells (Figures 2(a) and 2(b)). The colony formation assay results revealed that lnc-ZNF281 overexpression inhibited cell colony formation in glioma cells (Figures 2(c) and 2(d)). These results suggested that overexpression of lnc-ZNF281 inhibited the proliferation of glioma cells *in vitro*. We next performed wound-healing assay and transwell assay to explore the effect of lnc-ZNF281 on the migration of glioma cells. The wound-healing assay results showed that overexpression of lnc-ZNF281 inhibited the migrative ability of glioma cells significantly (Figures 3(a) and 3(b)). Similar results were obtained from the transwell assay (Figures 3(c) and 3(d)).

### 3.3. Inhibition of lnc-ZNF281 Promoted the Proliferation and Migration of Glioma Cells

**Figure 4:**
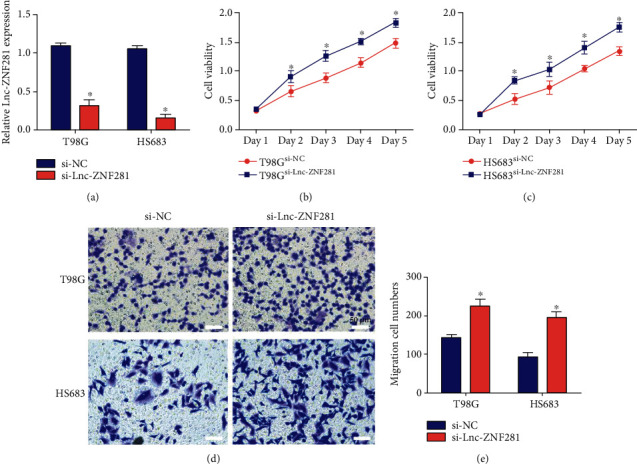
Inhibition of lnc-ZNF281 inhibited glioma cell proliferation and migration. (a) qRT-PCR results indicated that the expression level of lnc-ZNF281 in glioma cells decreased significantly after si-lnc-ZNF281 transfection. (b, c) The CCK-8 assay results indicated that inhibition of lnc-ZNF281 promoted the proliferation of glioma cells. (d, e) The transwell assay results indicated that overexpression of lnc-ZNF281 inhibited cell migration in glioma. Scale bars = 50 *μ*m. ^∗^*p* < 0.05.

si-lnc-ZNF281 was transfected into T98G and HS683 glioma cells to construct lnc-ZNF281 silencing glioma cell lines (Figure 4(a)). After that, we performed CCK-8 and transwell assays to explore the effect of lnc-ZNF281 downregulation on glioma cell proliferation and migration. The CCK-8 assay results showed that inhibition of lnc-ZNF281 promoted the proliferative ability of glioma cells (Figures 4(b) and 4(c)). The transwell assay results showed that lnc-ZNF281 downregulation enhanced the migrative ability of glioma cells (Figures 4(d) and 4(e)).

### 3.4. Overexpression of lnc-ZNF281 Inhibited the AKT/GSK-3*β*/*β*-Catenin Pathway in Glioma Cells

**Figure 5:**
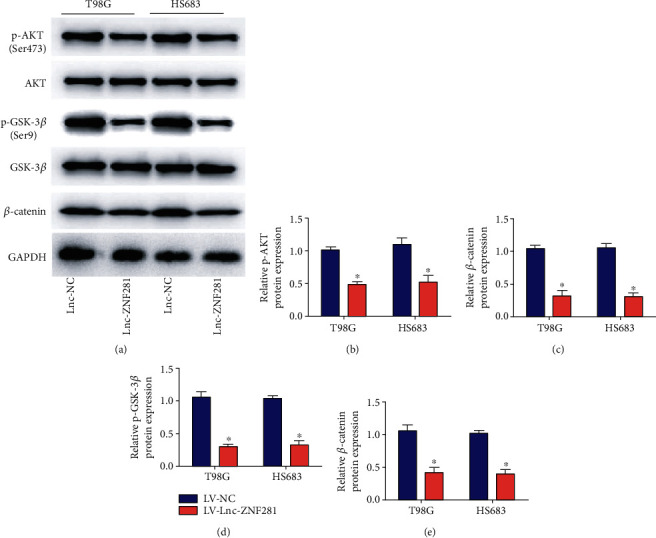
Overexpression of lnc-ZNF281 inhibited the AKT/GSK-3*β*/*β*-catenin pathway in glioma cells. (a–c) The Western blot results showed that overexpression of lnc-ZNF281 decreased the protein expression levels of p-AKT and p-GSK-3*β*, while had no effect on total AKT and GSK-3*β* protein levels. (d, e) Results of Western blot and qPCR demonstrated that increased lnc-ZNF281 suppressed the expression of *β*-catenin mRNA and protein. ^∗^*p* < 0.05.

To further study the potential molecular mechanisms of lnc-ZNF281 affecting glioma cells, we detected the protein levels of some classical markers, like AKT, GSK-3*β*, and *β*-catenin, by Western blotting assay. qRT-PCR was performed to examine the expression level of *β*-catenin mRNA. The results showed that overexpression of lnc-ZNF281 decreased the protein expression levels of p-AKT and p-GSK-3*β*, while had almost no effect on total AKT and GSK-3*β* protein levels (Figures 5(a)–5(c)). lnc-ZNF281 overexpression decreased *β*-catenin mRNA and protein levels (Figures 5(d) and 5(e)). These results suggested that lnc-ZNF281 regulated the proliferation and migration of glioma cells through AKT/GSK-3*β*/*β*-catenin pathway.

### 3.5. *β*-Catenin Agonist Reversed the Effects of lnc-ZNF281 Upregulation on Glioma Cells

**Figure 6:**
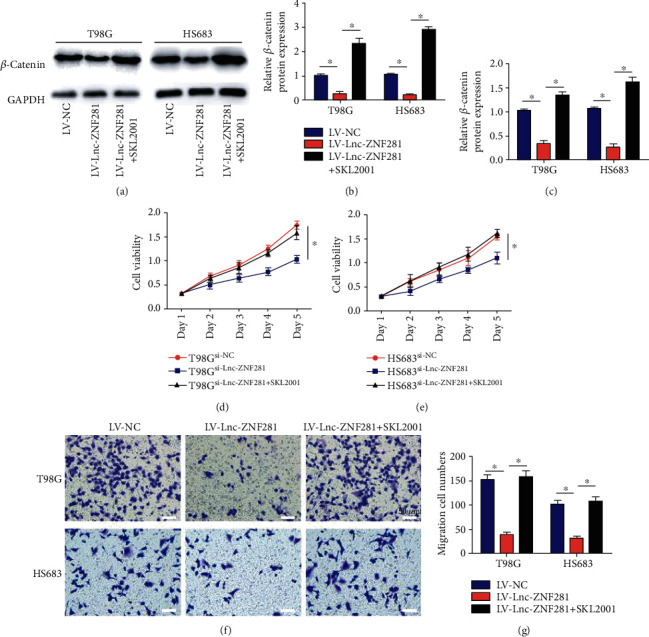
Increased *β*-catenin reversed the suppressive effects of lnc-ZNF281 on glioma cells. (a–c) *β*-catenin agonist SKL2001 was used to upregulate *β*-catenin expression, and the Western blot results showed that SKL2001 significantly increased the protein expression levels of *β*-catenin. (d, e) The CCK-8 assay results indicated that *β*-catenin reversed the inhibitory effects of lnc-ZNF281 on the proliferation of glioma cells. (f, g) The transwell assay results indicated that *β*-catenin reversed the inhibitory effects of lnc-ZNF281 on the migration of glioma cells. Scale bars = 50 *μ*m. ^∗^*p* < 0.05.

To further investigate whether lnc-ZNF281 suppressed glioma cell proliferation and migration via AKT/GSK-3*β*/*β*-catenin signaling pathway, we treated lnc-ZNF281 overexpressing glioma cells with *β*-catenin agonist SKL2001 for 30 min [17]. After that, the mRNA and protein levels of *β*-catenin were upregulated (Figures 6(a)–6(c)). Further functional assays showed that glioma cell proliferation and migration were reversed after treated with *β*-catenin agonist SKL2001, compared with lnc-ZNF281 overexpression alone (Figures 6(d)–6(g)). These findings confirmed the conjecture that lnc-ZNF281 suppresses glioma cell proliferation and migration by regulating AKT/GSK-3*β*/*β*-catenin pathway.

## 4. Discussion

lncRNA, as a research hotspot in recent years, has been confirmed to be closely related to the biological process of many tumors, including gastric cancer, breast cancer, liver cancer, and colorectal cancer [18–20]. As a novel lncRNA identified recently, lnc-ZNF281 has been reported to be associated with many tumors. For instance, lnc-ZNF281 decreased miRNA-124 level to promote the migration and invasion of gastric cancer cells [16]. Knockdown of lnc-ZNF281 inhibited colorectal cancer cell proliferation, migration, and invasion via the Wnt/*β*-catenin pathway [21]. Moreover, by activating the Wnt/*β*-catenin pathway, lnc-ZNF281 promoted cell proliferation and invasion in pancreatic cancer cells [22]. On the other hand, glioma is one of the most common malignant tumors in brain tumors, which has a high recurrent rate after surgical resection. Many lncRNAs could regulate the progression of glioma, like NEAT1, HOTAIR, and GAS5 [23–25]. However, there are no reports that revealed the relationship between lnc-ZNF281 expression and the progression of glioma up to date. The role of lnc-ZNF281 in regulating glioma and the potential molecular mechanism might provide a novel direction in the study of glioma occurrence and development.

In our study, we extracted total RNAs from 30 glioma tissues and 22 normal brain tissues. Then, we detected the expression level of lnc-ZNF281 using qRT-PCR. Results showed that lnc-ZNF281 had a low expression in glioma tissues. For exploring the role of lnc-ZNF281 in glioma, we transfected T98G and HS683 cells with lentiviral vectors and siRNAs to structure stably transfected glioma cell lines, which was confirmed by qRT-PCR. Then, a series of experiments were performed to investigate whether lnc-ZNF281 could regulate the progression of glioma cells. The cell proliferation results revealed that overexpression of lnc-ZNF281 inhibited cell proliferation in both T98G and HS683 cells. The wound-healing assay and transwell assay results showed that overexpression of lnc-ZNF281 repressed the migrative abilities of glioma cells. Moreover, silencing lnc-ZNF281 obtained the opposite findings. Consequently, these results above demonstrated that dysregulation of lnc-ZNF281 does regulate the biological process of glioma cells, which is worthy of further study.

Many studies indicate that changes of signaling pathways are closely related to the occurrence of glioma [10, 26], while dysregulation of lncRNAs causes abnormal expression of some key factors to change the activity of corresponding signaling pathways, subsequently to regulate the biological process of glioma. For example, dysregulation of PI3K/AKT signaling pathway enhances the occurrence and progression of glioma [27]. Sensitization of the Wnt/*β*-catenin pathway promotes cell proliferation, migration, and angiogenesis in glioma [28]. Interestingly, numerous downstream protein substrates are activated after the phosphorylation of AKT, including GSK-3*β*, which is known as the degradation factor of *β*-catenin [29]. Additionally, GSK-3*β* regulates the stability of *β*-catenin protein directly to influence Wnt/*β*-catenin signaling pathway [30]. Besides, lnc-ZNF281 regulates the Wnt/*β*-catenin pathway to influence the progression of colorectal cancer [21]. We wonder that whether lnc-ZNF281 suppressed the progression of glioma by AKT/GSK-3*β*/*β*-catenin pathway. We performed Western blot and qRT-PCR assays to measure the levels of p-AKT, AKT, p-GSK-3*β*, GSK-3*β*, and *β*-catenin. The results of Western blot and qRT-PCR showed that overexpression of lnc-ZNF281 inhibited phosphorylation of AKT and GSK-3*β* and decreased *β*-catenin mRNA and protein levels. These results suggested that lnc-ZNF281 regulated the progression of glioma via AKT/GSK-3*β*/*β*-catenin signaling pathway.

The importance of lncRNAs in diagnosis and treatment has been reported in glioma and numerous other cancers [11, 12, 31]. Here, we found that lnc-ZNF281 suppressed glioma cell proliferation and migration and downregulated AKT/GSK-3*β*/*β*-catenin signaling pathway. However, the research is in a very early stage and lacks animal and clinical experiments. It will be of significance to further test the effects of lnc-ZNF281 in the treatment of human glioma. Besides, there are some limitations in our study. First, the numbers of glioma tissues and normal brain tissues are insufficient. More clinical tissues should be used to confirm the expression levels of lnc-ZNF281 in glioma tissues and cell lines. Second, the expression level of lnc-ZNF281 in blood samples should be detected to explore the potential to be a biological marker for diagnosis and treatment.

## 5. Conclusions

Our study confirmed the downregulation of lnc-ZNF281 in glioma tissues. Moreover, we revealed that lnc-ZNF281 might inhibit proliferation and migration in glioma via AKT/GSK-3*β*/*β*-catenin signaling pathway. Therefore, lnc-ZNF281 might become a novel direction for the research of biological progression of glioma, and it may be an effective target for glioma patients.

## Data Availability

Data in our study can be acquired by emailing the corresponding author upon reasonable request.
